# Progress and Prospects in FRET for the Investigation of Protein–Protein Interactions

**DOI:** 10.3390/bios15090624

**Published:** 2025-09-19

**Authors:** Yue Zhang, Xinyue Ma, Meihua Zhu, Vivien Ya-Fan Wang, Jiajia Guo

**Affiliations:** 1 Institute of Biomedical and Health Engineering, Shenzhen Institutes of Advanced Technology, Chinese Academy of Sciences, Shenzhen 518055, China; yue.zhang2@siat.ac.cn (Y.Z.); xy.ma6@siat.ac.cn (X.M.); mh.zhu@siat.ac.cn (M.Z.); 2 Faculty of Health Sciences, University of Macau, Avenida da Universidade, Taipa, Macau SAR, China; 3 Cancer Centre, Faculty of Health Sciences, University of Macau, Avenida da Universidade, Taipa, Macau SAR, China; 4 MoE Frontiers Science Center for Precision Oncology, University of Macau, Avenida da Universidade, Taipa, Macau SAR, China

**Keywords:** FRET, protein–protein interactions

## Abstract

Protein–protein interactions (PPIs) play a crucial role in various biological processes, including signal transduction, transcriptional regulation, and metabolic pathways. Over the years, many methods have been developed to study PPIs, such as yeast two-hybrid (Y2H), co-immunoprecipitation (Co-IP), pull-down assays, and surface plasmon resonance (SPR). However, each of these techniques has its own limitations, including false positives, a lack of specific binding partners, and restricted interaction zones. Fluorescence resonance energy transfer (FRET) has emerged as a powerful technique for investigating PPIs, offering several advantages over traditional methods. Recent advancements in fluorescence microscopy have further enhanced its application in PPI studies. In this review, we summarize recent developments in FRET-based approaches and their applications in PPIs research over the past five years, including conventional FRET, time-resolved FRET (TR-FRET), fluorescence lifetime imaging microscopy-FRET (FLIM-FRET), single-molecule FRET (smFRET), fluorescence cross-correlation spectroscopy FRET (FCCS-FRET), and provide guidance on selecting the most appropriate method for PPIs studies.

## 1. Introduction

Proteins are highly versatile macromolecules that not only catalyze essential biochemical reactions but also provide structural integrity to cells. Their diverse functions often depend on the ability to form complexes with other proteins and/or to undergo allosteric conformational changes, thereby facilitating numerous intracellular processes [1]. Among these functional interactions, protein–protein interactions (PPIs) play an important role in regulating key biological activities, including cell signaling, metabolic pathways, immune responses, and disease progression. PPIs are fundamental to intracellular and intercellular communication, influencing processes such as cell growth, differentiation, apoptosis, and homeostasis [2]. They are also essential for maintaining cellular architecture, as structural proteins interact to preserve cell shape and mechanical stability. Particularly, membrane proteins contribute to the integrity of the cell membrane and participate in signal transduction and molecular transport. Many membrane proteins function as receptors, binding to extracellular ligands such as hormones or neurotransmitters to activate downstream signaling cascades that govern cellular physiology [3]. In the context of the immune system, PPIs enable cell surface recognition, mediate antigen–antibody interactions, and coordinate cytokine–receptor binding, all of which are vital for mounting effective immune responses [4]. Moreover, PPIs are integral to gene regulation, where transcription factors interact with co-activators, repressors, or other regulatory proteins to fine-tune gene expression in response to environmental and developmental signals [5]. Disruptions in PPIs can lead to pathological conditions. For instance, aberrant interactions may drive uncontrolled cell proliferation in cancer [6], or result in protein aggregation—such as amyloid-β and tau-which are hallmarks of neurodegenerative diseases like Alzheimer’s and Parkinson’s [7]. Monitoring these interactions offers valuable early diagnostic insights. Furthermore, identifying aberrant PPIs may uncover biomarkers for infectious and chronic diseases and guide the development of targeted therapeutics [8]. Given the central role of PPIs in health and disease, they have become a focal point for pharmaceutical intervention. By modulating PPIs—either by disrupting pathological interactions or enhancing beneficial ones—researchers can develop novel therapeutic strategies for a wide range of diseases [9]. Thus, understanding and characterizing PPIs is critical not only for elucidating biological mechanisms but also for advancing diagnostics and therapeutic development.

**Figure 1:**
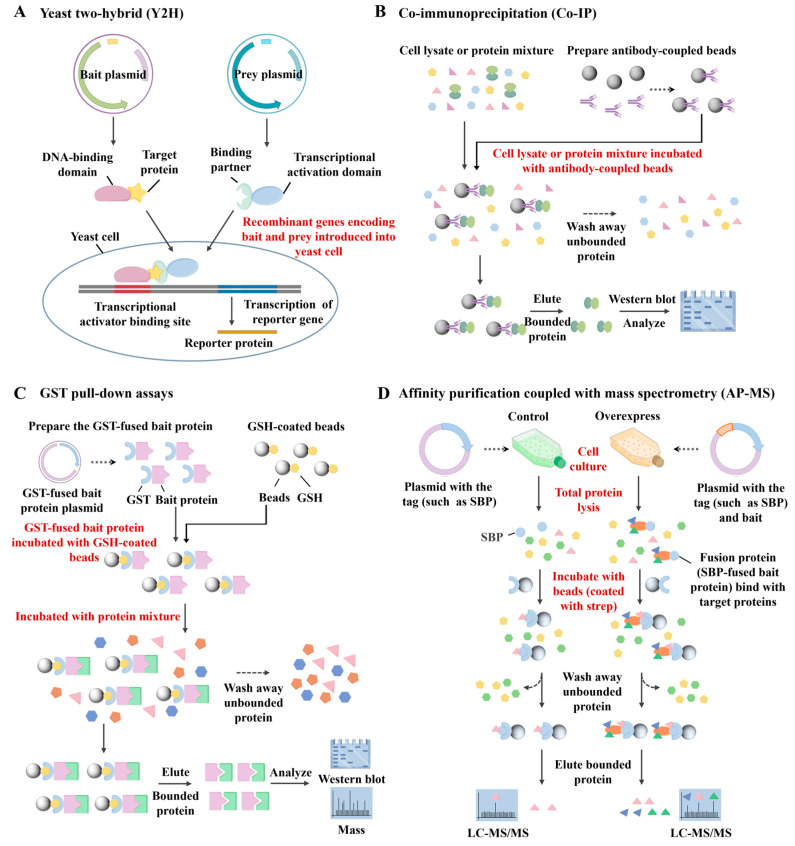
Schematic diagram of (**A**) the principle of yeast two-hybrid (Y2H) assays, (**B**) co-immunoprecipitation (Co-IP), (**C**) pull-down assays, and (**D**) affinity purification coupled with mass spectrometry (AP-MS).

In recent years, a variety of techniques have been employed to investigate PPIs, including yeast two-hybrid (Y2H) assays [10], co-immunoprecipitation (Co-IP) [11], pull-down assays [12], and affinity purification coupled-mass spectrometry (AP-MS) [13], among others. Each of these methods offers specific advantages but also suffers from inherent limitations. For example, the Y2H assay (Figure 1A) identifies PPIs by fusing proteins of interest to the DNA-binding and activation domains of the GAL4 transcription factor. When the two proteins interact, the functional reconstitution of GAL4 drives reporter gene expression. Although widely used, Y2H is limited by a high rate of false positives, inability to detect interactions involving membrane proteins or cytoplasmic complexes, and a bias toward proteins that localize to the nucleus [14]. Co-IP (Figure 1B) enables detection of PPIs under near-physiological conditions by isolating protein complexes from cell lysates using specific antibodies. This technique preserves post-translational modifications and native interaction contexts. However, it often captures indirect interactions within large protein complexes, has limited sensitivity, and may yield false negatives due to transient or weak binding events [15]. Pull-down assays (Figure 1C), an in vitro technique similar to Co-IP, use tagged bait proteins to capture binding partners via affinity purification. The resulting complexes are typically analyzed by SDS-PAGE, Western blotting, or mass spectrometry. While useful, this approach lacks the capability to capture dynamic interactions within living cells [16,17]. AP-MS (Figure 1D) leverages epitope-tagged proteins as baits to isolate their interaction partners. This method provides high sensitivity and can detect multiple interactors simultaneously. However, it faces challenges in capturing membrane-associated proteins, and often struggles with discriminating direct from indirect interactions or retaining weak/transient associations [18,19]. We compare the advantages and limitations of PPI technologies in Table 1.

Given the limitations of conventional techniques, Förster resonance energy transfer (FRET) has become a powerful method for studying PPIs in real time and under physiological conditions [20]. First described by Theodor Förster in 1948 [21], FRET is a non-radiative energy transfer process that occurs through dipole–dipole coupling between a donor and an acceptor [22]. Crucially, the efficiency of this energy transfer is exquisitely sensitive to the distance between the fluorophores, typically effective within a range of 1–10 nanometers, known as the Förster radius [23]. This high distance sensitivity makes FRET particularly well-suited for PPI research, as proteins—although structurally diverse—generally have diameters below 10 nm, allowing FRET to detect direct molecular interactions with nanometer-scale precision. Acting as a “molecular ruler”, FRET enables the visualization of interaction dynamics that are difficult to resolve using conventional biochemical methods. In addition, FRET offers high spatiotemporal resolution and functions reliably in live-cell environments, providing sensitive and accurate readouts that are resistant to background noise and environmental fluctuations [24]. It is worth noting that BRET (Bioluminescene resonance energy transfer) is also used to study PPIs. Unlike FRET, BRET uses bioluminescence as the energy source and does not require an external excitation light, which can reduce photodamage. It is widely used in PPI studies, especially for membrane proteins. For instance, Besson et al. propose a BRET saturation method that facilitates the PPI quantification in living cells [25].

In the past five years, FRET-based technologies have been extensively applied in PPI research, yielding substantial advancements in methodology and application scope. For example, Yang et al. employed conventional FRET to simultaneously monitor the formation of heterotrimeric complexes among Bad, Bcl-xL, and tBid in mitochondria, demonstrating its utility in studying PPI stoichiometry and affinity within the Bcl-2 apoptotic signaling pathway [26]. Conventional FRET is the most used form based on steady-state fluorescence intensity measurements between a donor and an acceptor fluorophore. However, its accuracy can be affected by background fluorescence and spectral crosstalk.

To overcome these limitations, time-resolved FRET (TR-FRET) was developed. Unlike intensity-based FRET, TR-FRET detects interactions by measuring changes in the fluorescence lifetime, and is independent of fluorophore concentration. By utilizing long-lifetime probes (e.g., lanthanide chelates) and a time-gated detection technique, TR-FRET effectively eliminates background signals, significantly enhancing detection sensitivity. This makes it especially suitable for low-abundance target detection. Tang et al. established a TR-FRET–based screening protocol for PPI modulators, which remains effective even at low protein concentrations and holds promise for discovering small-molecule PPI inducers and inhibitors [27].

Besides TR-FRET, another fluorescence lifetime-based approach is fluorescence lifetime imaging microscopy FRET (FLIM-FRET), which enables direct visualization of PPIs with high temporal and spatial resolution by allowing spatially resolved imaging of molecular interactions within live cells and tissues. Khramtsov et al. employed FLIM-FRET to visualize the subcellular distribution and dynamic behavior of Keap1 in live cells, revealing interaction features that could not be resolved using intensity-based methods [28].

Although FLIM-FRET is suitable for population-level imaging, single-molecule FRET (smFRET) provides extremely high spatial and temporal resolution at the individual molecule level. This makes it ideal for studying molecular mechanisms, conformational changes, and PPI kinetics in real time [29]. Chen et al. used smFRET to observe a continuum of NF-κB conformations in both free and DNA-bound states, revealing structural transitions that occur on a timescale from subseconds to minutes-comparable to physiological DNA-binding events [30].

While conventional smFRET typically requires in vitro environments and the involvement of sample immobilization, these conditions may disrupt the native state of protein interactions. To investigate PPIs at the single-molecule level in live cells, a more suitable approach is fluorescence cross-correlation spectroscopy combined with FRET (FCCS-FRET). This technique enables quantitative analysis of molecular interactions by monitoring the correlated diffusion of two fluorescently labeled molecules within a femtoliter-scale observation volume, providing information on individual molecular concentration and dynamics. For example, Shi et al. analyzed the conformational features of ephrin, a receptor tyrosine kinase type-A receptor 2 (EphA2), within live monkey kidney cells based on pulsed interleaved excitation–FCCS FRET (PIE-FCCS-FRET) [31].

**Figure 2:**
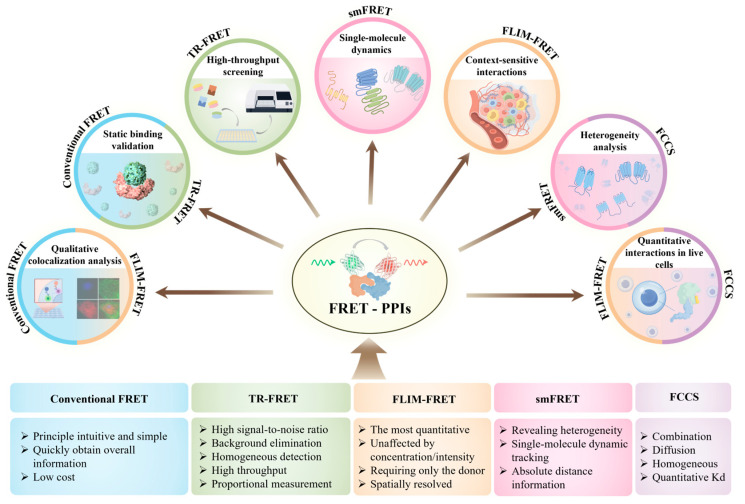
FRET techniques and their applications in PPI studies. The diagram illustrates five major FRET-based methods and their representative applications in PPI research. The width of each colored square (**bottom**) reflects the relative number of studies using that technique. Color matches between squares and application fields (**top**) indicate which techniques are applicable to specific biological contexts.

A comparative overview of these FRET variants is presented in the following sections to assist researchers in selecting the most appropriate technique based on specific experimental requirements. Figure 2 summarizes the key features of commonly used FRET methods and highlights their biological applications in PPI research, offering a visual reference for understanding their respective strengths, limitations, and use cases.

## 2. FRET Technology

**Figure 3:**
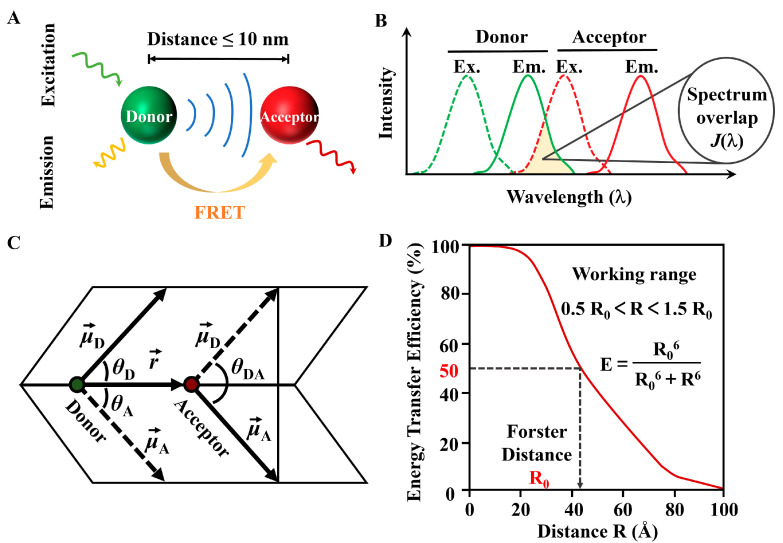
(**A**) Schematic of the FRET principle. (**B**) Overlap integral for donor fluorescence and acceptor excitation. (**C**) The calculation parameters of the FRET dipole factor κ. (**D**) FRET-efficiency over distance.

FRET has attracted the attention of researchers across various disciplines as a powerful tool, including basic life sciences, theoretical physics, chemistry, and applied technologies in physics, electronics, medicine, and biology. FRET is a nonradiative energy transfer that occurs through dipole coupling from an excited state donor (D) fluorophore to a ground state acceptor (A) [32]. The FRET phenomenon requires the following conditions to be met, the distance between the donor and acceptor molecules must be sufficiently close, typically within the range of 1–10 nanometers (Figure 3A), and up to ~20 nanometers for special FRET pairs, and there must be sufficient overlap between the emission spectrum of the donor and the absorption spectrum of the acceptor (Figure 3B) [33], the dipole orientations of the donor and acceptor molecules need to be appropriate; for example, the angle between the donor emission transition dipole moment and the acceptor absorption transition dipole moment cannot be 90 degrees; otherwise, orientation factor $\kappa^{2}$ is 0, and the FRET efficiency is 0 (Figure 3C). The value of $\kappa^{2}$ ranges from 0 to 4; if the dipole moment of the acceptor is perpendicular to the electric field of the donor, then $\kappa^{2}$ is 0, resulting in a FRET efficiency of zero. It is important to briefly outline its basic principles, which make it sensitive in determining molecular distances and revealing molecular complexes. Below are some commonly used formulas in this technology; the first is the FRET efficiency ($E_{FRET}$).

(1) $$E_{FRET}=1/\left[1+{\left(r/R_{0}\right)}^{6}\right]=k_{T}/\left(k_{T}+1/\tau_{D}\right)$$

where r is the distance between the donor and acceptor, and $R_{0}$ is the Förster distance—the distance at which the transfer efficiency is 50% (Figure 3D); $k_{T}$ is the rate of energy transfer; $\tau_{D}$ is the lifetime of the donor excited state in the absence of the acceptor. This equation shows that the FRET efficiency decreases sharply with increasing distance. Calculating $R_{0}$ and measuring $E_{FRET}\;$ provides r, the common descriptive factor of FRET as a molecular-scale ruler.

(2) $${R_{0}}^{6}=0.021J\kappa^{2}\Phi_{D}n^{4}$$

where $\kappa^{2}$ is the orientation factor; there are certain averaging conditions that offer a reliable approximation for $\kappa^{2}$. In many practical FRET applications, when both the donors (D) and acceptors (A) can adopt any orientation during the FRET process, meaning the average rotation rate is much greater than the average FRET rate, the system enters a dynamic averaging regime, and $\kappa^{2}$ is approximated as 2/3. Additionally, n is the refractive index of the medium, $\Phi_{D}$ is the quantum yield of the donor fluorescence in the absence of acceptor, and J is the overlap integral between the emission spectrum of the donor and absorption spectrum of the acceptor.

(3) $$J=\int \bar{I_{D}}\varepsilon_{A}\lambda^{4}d\lambda$$

The spectral overlap between donor emission and acceptor absorption provides the energetic resonance that is necessary for FRET. J is quantified by the integral of the normalized donor emission intensity, $\varepsilon_{A}$ is the molar extinction coefficient of the acceptor (units M^−1^cm^−1^), and λ is the wavelength. The donor requires good spectral overlap, while the acceptor needs a large extinction coefficient. $\lambda^{4}$ indicates that as wavelengths increase, the overlap integral also rises. Thus, FRET pairs characterized by broad spectral overlap and high extinction coefficients in the acceptor can lead to larger overlap integrals.

Designing a FRET experiment requires careful planning and consideration of several key factors [34]. One critical aspect is selecting an effective labeling strategy, which involves identifying optimal donor–acceptor (D-A) positions in a structural model. D-A pairs can be introduced through various methods, including chemical labeling of native proteins, oligonucleotides, peptides, or recombinant fluorescent protein fusions. The choice of labeling method significantly impacts the FRET system. For instance, homogeneous labeling ensures consistent D-A distances, while heterogeneous labeling results in variable distances, which can lead to skewed results. Site-specific labeling of cysteine residues provides fixed D-A positions, whereas lysine labeling creates a distribution of positions. Another important consideration is selecting an effective D-A pair. This requires compatibility with the chosen labeling strategy, as well as alignment of $R_{0}$ and $E_{FRET}$ with the structural model and available instrumentation. The practicality of the chemistries and purification steps for labeling the target biomolecules is also essential. Key characteristics of a good D-A pair include a high $\varepsilon_{A}$, minimal direct excitation of the acceptor at the donor’s wavelength, and good spectral separation between their emission spectra. Selecting the appropriate instrumentation is equally crucial. Key considerations include determining the spatial resolution needs using cuvette or microtiter plate measurements for lower resolution or FRET imaging for higher resolution. Additionally, choosing between lifetime measurements and intensity measurements is important. Lifetime measurements are generally more robust in challenging backgrounds, as they are unaffected by photobleaching. It is also essential to ensure that the instrumentation aligns with the required excitation and emission wavelengths. Filter-based systems typically offer greater sensitivity, while spectral measurements better manage crosstalk. Minimum requirements include selective donor excitation and adequate resolution of donor and acceptor emissions. Based on the above principles, there are various methods to measure the spectral changes caused by FRET. These can be categorized into fluorescence intensity measurements, fluorescence lifetime measurements, and fluorescence anisotropy measurements. In the following sections, we will focus on advancements in FRET techniques based on these principles and their applications in studying PPIs.

The selection of fluorescent probes is also very important, as it directly determines the sensitivity, specificity, and reliability of FRET experiments [35]. Ideal probes should have good spectral compatibility, photostability, and biocompatibility. Excellent photostability means that the probe can resist photobleaching under prolonged illumination; this is crucial for live-cell imaging. Great biocompatibility means that for in vivo applications, the probe must exhibit minimal cytotoxicity and avoid interfering with the PPIs of target biomolecules [36]. Common probe types include organic dyes (such as Cy3/Cy5, Alexa Fluor pairs), fluorescent proteins (such as GFP/YFP, mTurquoise2/sYFP2), and quantum dots, each with unique advantages. For instance, organic dyes have high brightness and narrow emission spectra, but require chemical conjugation. Fluorescent proteins can be genetically encoded, but their spectra may be broader. Quantum dots have a broad excitation spectrum, a narrow emission spectrum, and a long fluorescence lifetime, but their synthesis is relatively complex. For in vitro studies, small organic dyes are preferred owing to their high brightness and narrow emission spectra. For in vivo applications, fluorescent proteins with high biocompatibility, photostability, and minimal immunogenicity should be chosen; the choice depends on your experimental requirements. Besides, setting up appropriate control experiments is also crucial, especially when researching with large proteins, as labeling of the donor and acceptor may interfere with protein function or related signaling pathways. For instance, such interference may alter a protein’s conformational changes and its responsiveness to stimuli; if the labeling site is close to phosphorylation sites, it may also decrease the activation efficiency of downstream effector molecules. Protein function can be verified through activity assays or ligand-binding experiments. Additionally, it is necessary to label the donor or acceptor separately to examine the impact of single labeling on the protein. Meanwhile, a non-interacting control should be established using known non-interacting proteins to rule out any possible interferences from non-specific energy transfer. The effect of labeling sites on protein conformation can also be predicted using molecular docking or dynamic simulation techniques.

## 3. FRET Technologies for the Investigation of PPIs

### 3.1. Conventional FRET

**Figure 4:**
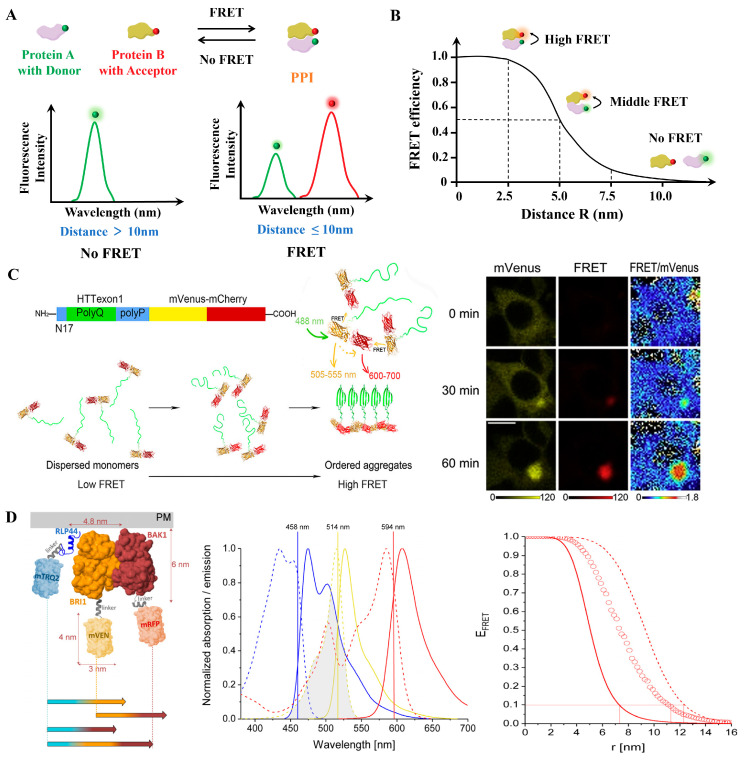
(**A**) The conventional FRET technique employed by PPIs research measures the non-radiative energy transfer from the donor to the receptor; the donor signal decreases while the receptor signal increases. (**B**) Dependency of FRET efficiency on the distance between the FRET pair (R). (**C**) A FRET-based method for monitoring structural transitions in protein self-organization. Reproduced from [44] with permission. Copyright 2022, Elsevier. (**D**) Three-fluorophore FRET analysis of ternary protein association in living plant cells. Schematic diagram of protein design for three-fluorescence FRET, normalized absorption (dotted lines) and emission (solid lines) spectra overlaps, and FRET efficiency spectrum. Adapted from [46] with permission. Copyright 2022, MDPI.

Conventional FRET relies on detecting changes in fluorescence intensity between donor and acceptor fluorophores to infer molecular proximity (Figure 4A,B). Its principle is relatively simple and does not require specialized equipment, making it widely accessible and cost-effective for large-scale or global analyses of PPIs [37,38,39]. Recent advances in fluorescent dyes, quantum dots, and genetically encoded tags have significantly enhanced the brightness and photostability of FRET probes, thereby improving detection sensitivity and measurement accuracy [40]. For example, Hoshino et al. developed a FRET probe by fusing fluorescent proteins (FPs) to a single protocadherin molecule, successfully visualizing calcium-dependent homophilic interactions in neurons [41]. When two fusion proteins interact, the donor and acceptor fluorophores come into proximity, generating a spatiotemporally resolved FRET signal that reflects the binding event [42]. Beyond neuronal signaling, conventional FRET has also been adapted for high-throughput drug screening platforms. Sowa et al. developed a cost-effective high-throughput screening platform based on this principle to identify small-molecule modulators of tankyrase PPIs [43]. In addition to screening applications, it has been used to monitor fundamental processes such as protein self-assembly. Wan et al. monitored protein self-assembly by fusing both donor and acceptor fluorophores to the same protein, enabling precise intermolecular FRET measurements (Figure 4C) [44]. These methodological optimizations further enable real-time monitoring of receptor dynamics. Han et al. further optimized FRET signal quality by systematically varying linker lengths and FP combinations to monitor estrogen receptor dynamics in real time [45]. Conventional FRET has also evolved to support multiplexed interaction analysis using different donor–acceptor pairs. This allows for simultaneous monitoring of multiple PPIs within complex signaling networks. For example, Glöckner et al. implemented a three-fluorophore FRET system using genetically encoded fluorophores mTurquoise2, mVenus, and mRFP in plant cells. Their study revealed that Receptor-Like Protein 44, BRI1, and BAK1 form a ternary complex within specific plasma membrane nanodomains, providing new insights into brassinosteroid signaling (Figure 4D) [46]. Additionally, conventional FRET has also become an indispensable tool in drug discovery, where it is used to screen potential therapeutic compounds, evaluate their effects on specific PPIs, and inform the development of targeted therapies for diseases such as cancer [47]. Moreover, FRET offers unique capabilities for probing protein conformational states, thereby advancing our understanding of how molecular interactions influence protein structure and function [48].

It cannot be ignored that conventional FRET suffers from critical limitations. First, spectral crosstalk and direct acceptor excitation can introduce false-positive signals. Uncertainty in the orientation factor further leads to distance measurement errors, which can be partially mitigated using rigid probes. Its spatial resolution also falls short for analyzing subcellular microdomains. For live-cell applications, phototoxicity poses a major challenge.

Overall, conventional FRET remains an essential and versatile tool in PPI research. It has been widely adopted in drug discovery to screen candidate compounds, evaluate their effects on target interactions, and develop targeted therapies for diseases such as cancer [47]. Moreover, it enables researchers to probe protein conformational dynamics, offering valuable insight into the structural and functional consequences of molecular interactions [48]. Looking ahead, improving the photostability of FRET probes and developing advanced theoretical models will be essential to reduce background interference and improve the signal-to-noise ratio [49]. In parallel, the design of novel fluorescence reporters that target specific types of PPIs is being prioritized. These customized probes can optimize donor–acceptor pairing, minimize non-specific interactions, and ensure appropriate spatial orientation, thereby enhancing measurement accuracy [50].

### 3.2. Time-Resolved FRET

**Figure 5:**
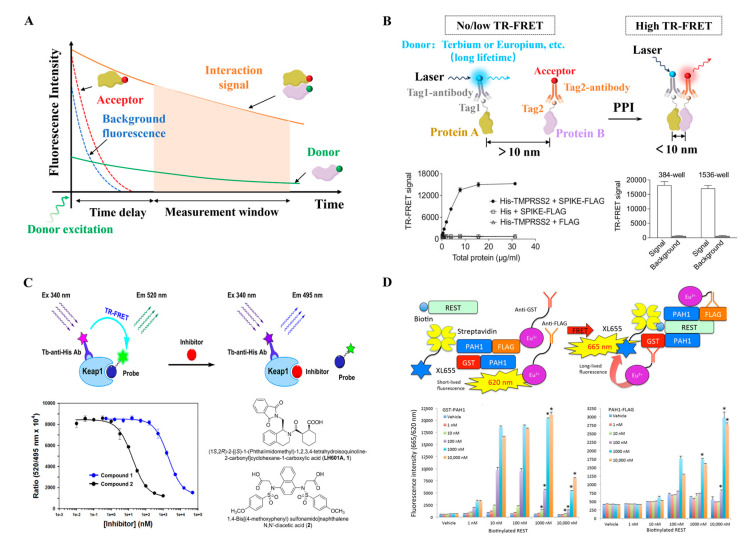
(**A**) In TR-FRET, detection after a short delay (typically 50–150 µs) post-excitation minimizes non-specific background fluorescence interference. (**B**) Schematic illustration of the design of the TR-FRET assay for monitoring PPIs. Adapted from [54] with permission. Copyright 2023, Oxford University Press. (**C**) Principle of a homogenous TR-FRET assay for the inhibition of Keap1–Nrf2 PPI, Terbium (Tb) serves as the donor and has a maximum emission peak at approximately 495 nm; fluorescein is used as the acceptor and has a maximum emission peak at 520 nm. Reproduced from [59] with permission. Copyright 2021, Elsevier. (**D**) Principle of TR-FRET assay mimicking PPI between PAH1 and REST, TR-FRET using GSTPAH1, PAH1-FLAG, biotinylated REST, antibodies-Eu3+, and XL665. * considered to be statistically significant if *p* < 0.00005. Reproduced from [61] with permission. Copyright 2021, MDPI.

As mentioned above, intensity-based FRET makes it highly susceptible to interference from background fluorescence, autofluorescence, and spectral crosstalk. These issues can reduce signal accuracy and compromise the reliability of experimental results, particularly in complex biological samples where non-specific signals are prevalent. Rather than relying on raw intensity, TR-FRET collects emission signals after a defined delay following excitation, effectively filtering out background fluorescence. This approach significantly enhances both signal specificity (Figure 5A) [51]. TR-FRET uses long-lifetime donors, organic ultralong room-temperature phosphorescence (OURTP) materials have advantages in TR-FRET. Yu et al. proposed a universal strategy to endow polymeric afterglow materials with wide color, ultralong lifetimes, and persistent near-infrared (NIR) emission. Their study guides the future design of NIR-emission OURTP materials [52].

TR-FRET is widely used in high-throughput screening due to its sensitivity, specificity, and compatibility with homogeneous, no-wash formats. Larsen et al. introduced a TR-FRET assay for screening compound libraries against rat sarcoma (RAS) kinases and rapidly accelerated fibrosarcoma (RAF) kinases PPIs. This assay facilitates the discovery of compounds that specifically target the GTP-bound active conformation of GTPase K-RAS [53]. Cicka et al. advanced this approach by developing an ultra-high throughput screening TR-FRET assay to monitor PPIs in cell lysates, facilitating the identification of small-molecule disruptors targeting disease-relevant PPIs [54] (Figure 5B). Funato et al. further expanded the use of TR-FRET by designing a screening system using long-lifetime donors and time-delayed detection to eliminate short-lived background fluorescence from media, proteins, or compounds. This enabled accurate PPI detection between phosphatase of regenerating liver (PRL) and cyclin M (CNNM) in physiological environments without the need for washing steps, making it ideal for automated high-throughput screening workflows [55].

Beyond high-throughput screening, TR-FRET has also been widely applied in drug discovery, particularly for identifying small-molecule modulators of PPIs [56]. Wang et al. developed a TR-FRET assay to evaluate inhibitors targeting the programmed cell death-1 (PD-L1)/programmed cell death-ligand 1 (PD-L1) interaction, leading to the discovery of a promising small-molecule modulator with an IC_50_ of 3.8 nM, which also demonstrated potent in vivo antitumor activity [57]. Similarly, Abed et al. and Lee et al. employed TR-FRET to identify potent inhibitors of the Kelch-like ECH-associated protein 1 (Keap1)–Nrf2 interaction. Nrf2 is the central transcription factor. Among the identified compounds, one featuring a C2-phthalimidopropyl group exhibited an IC_50_ of 2.5 nM, demonstrating strong inhibitory activity in cellular assays [58,59,60] (Figure 5C). These studies underscore the utility of TR-FRET in the discovery of PPI modulators.

In addition to screening and inhibitor discovery, TR-FRET can be integrated with complementary biophysical techniques or optimized sensor designs to further expand its utility, such as surface plasmon resonance (SPR), which could provide a comprehensive understanding of PPIs. Harada et al. demonstrated the feasibility of using both TR-FRET and SPR to study the interaction between SIN3 transcription regulator family member B (Sin3B) and repressor element-1 silencing transcription factor (REST), showcasing the potential of combining multiple technologies in PPI research [61] (Figure 5D). In addition to combining TR-FRET with other technologies, the study by Chen et al. demonstrated that optimizing sensor design combined with time-gated detection technology significantly improved the dynamic range and signal-to-noise ratio, highlighting its great potential in live-cell imaging and high-throughput screening [62]. In future research, by optimizing tag sequences and experimental conditions [54], and detecting dynamic differences [63], researchers will achieve efficient detection of PPIs [27].

TR-FRET is constrained by several key limitations. First, its homogeneous assay lacks spatial resolution, precluding the analysis of subcellular localization of interactions. Additionally, the use of large probes introduces steric hindrance, which can reduce signal intensity. Inner filter effects further pose challenges. At high sample concentrations, data distortion occurs, requiring either sample dilution or the adoption of dyes with wide Stokes shifts to mitigate this issue. Temporally, millisecond-scale resolution limits its ability to detect rapid kinetic processes.

### 3.3. Fluorescence Lifetime Imaging FRET

Beyond TR-FRET, another lifetime-based FRET technique is FLIM-FRET. FLIM-FRET extends the capability from bulk lifetime measurements to spatially resolved mapping of molecular interactions between proteins or signaling molecules within live cells and tissues. It enables real-time visualization of binding events, interaction dynamics, and signal transduction pathways with minimal disruption to cellular function.

**Figure 6:**
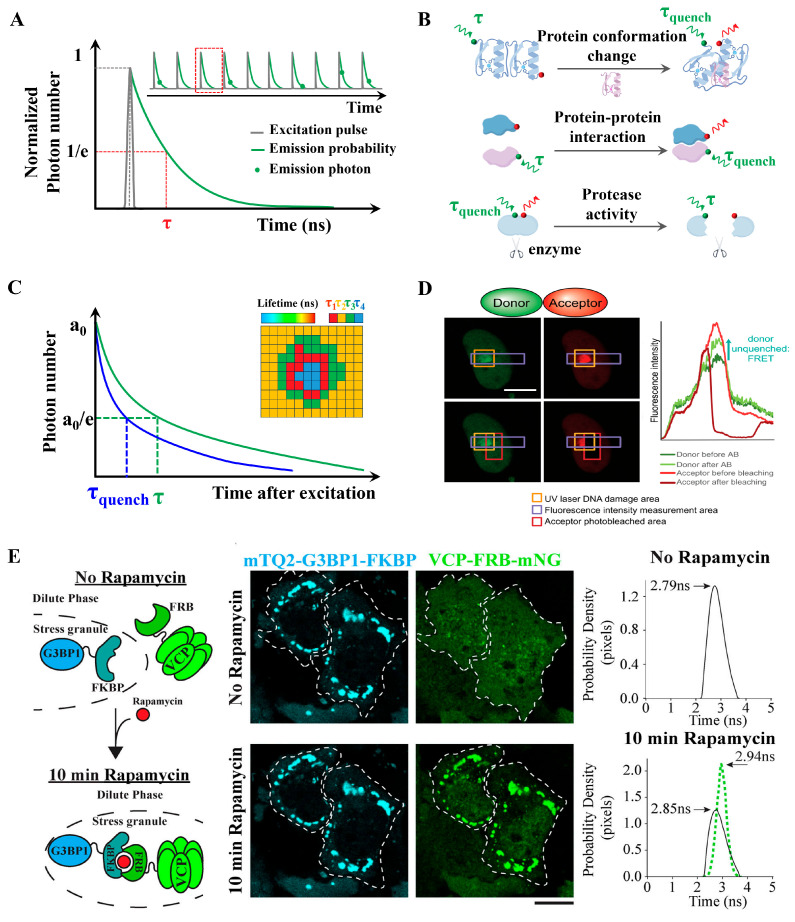
FLIM-FRET concept and applications. (**A**) Schematic of TCSPC. TCSPC FLIM acquisition includes the short excitation pulse, a single exponential fluorescence decay curve, and lifetime (τ) defined at the 1/e value. Inset shows detected single fluorescence photons (green dots) at different time periods within multiple excitation pulses. (**B**) Applications: Donor and acceptor pairs in separate molecules can be used to detect receptor/ligand binding; donor and acceptor pairs within the same molecule can be used to detect protein conformational changes or cleavage of proteins by proteases. (**C**) Lifetime τ shifts to shorter times due to FRET (τ_quench_) in FLIM-FRET analysis. Measuring the lifetime at each position in a scanning system yields a spatial map of lifetimes (Inset). (**D**) Direct measurement of PPIs by FLIM-FRET at UV laser-induced DNA damage sites in living cells. Damage sites were identified by H2AX immunofluorescence staining and by increased fluorescence intensity due to protein accumulation at the damage site (marked by an orange square). The acceptor was bleached in a region marked with a red square, and the intensity of mEGFP was measured across a region marked with a purple square before and after bleaching the acceptor. Reproduced from [65] with permission. Copyright 2020, Oxford University Press. (**E**) Fluorescence lifetime sorting reveals tunable enzyme interactions within cytoplasmic condensates. Cartoon representation, images, and fluorescence-lifetime histograms of the rapamycin-inducible FKBP and FRB dimerization system used to recruit VCP-FRBmNG to mTQ2-G3BP1-FKBP–enriched stress granules. Adapted from [69] with permission. Copyright 2025, Rockefeller University Press.

At the technical core of FLIM-FRET is time-correlated single-photon counting (TCSPC), a method that records the arrival time of individual fluorescence photons relative to the excitation pulse with nanosecond precision. TCSPC systems incorporate pulsed laser excitation and single-photon detectors, registering photon arrival times relative to the excitation pulse, thereby generating a fluorescence decay curve. This decay is typically modeled using a single-exponential function, allowing extraction of the fluorescence lifetime value (τ) (Figure 6A). By imaging and comparing fluorescence lifetimes across different regions, FLIM enables high temporal resolution measurements, allowing for real-time monitoring of lifetime changes in fluorescent molecules. These measurements provide critical information about molecular environments and interaction states. In addition, FLIM can simultaneously analyze the lifetimes of multiple fluorophores (Figure 6B), making it a non-invasive method well suited for live-cell imaging (Figure 6C) [64]. Lifetime data can be mapped across cells or tissues, and analyzed to determine the fraction of donor molecules undergoing FRET, from which FRET efficiency can be calculated. This enables inference of binding affinity, interaction kinetics, and conformational changes in molecular complexes.

FLIM-FRET is not affected by fluorescence intensity, photobleaching, or spectral crosstalk. FLIM recording is unaffected by variations in sensor expression levels, sensor bleaching, excitation fluctuations, and minor misfocusing. FLIM-FRET also avoids artifacts related to chromatic aberration and differences in sensitivity between channels, issues commonly encountered in ratio imaging (Figure 6D) [65,66]. Since FLIM is largely independent of protein concentration, it is particularly suitable for quantitatively analyzing PPIs in live cells [67]. For example, in the study of the Arabidopsis PII protein interaction network, FLIM-FRET was used to observe the dynamic formation and disappearance of PII foci, providing crucial spatiotemporal information for understanding the function of AtPII in chloroplasts [68]. Beyond plant systems, FLIM-FRET has also been adapted to monitor dynamic PPI networks in mammalian cells, such as ribonucleoprotein condensates. Fahim et al. developed an automated and rigorous FLIM-FRET-based method to track dynamic changes in PPIs within ribonucleoprotein (RNP) condensates in live cells, enabling visualization of interaction network dynamics under physiological conditions (Figure 6E) [69]. FLIM-FRET can detect transient or weak interactions, as well as monitor real-time conformational changes. Its multiplexing capability allows for the use of different donor–acceptor pairs simultaneously, enabling the study of multiple interactions within a single sample. For instance, Eckenstaler et al. employed a two-step acceptor photobleaching protocol in a three-fluorophore system to differentiate between non-interacting, dimeric, and trimeric interaction states [70]. Melle et al. used FLIM measurements to reveal that a calcium-binding protein interacts with multiple annexin family proteins in living cells [66].

In addition, FLIM-FRET enables visualization of the spatial distribution of protein interactions within cells, offering new insights into cellular signaling pathways. Khramtsov et al. utilized FLIM-FRET to study the intracellular distribution of modular nanotransporters containing monobodies targeting Keap1, demonstrating the ability to monitor intracellular trafficking of therapeutic agents [28]. Single-cell FLIM analysis also allows researchers to assess cell-to-cell heterogeneity in protein interactions, as opposed to averaging over populations. FLIM can detect PPIs in vivo at single-cell resolution and identify their subcellular localization. Two-photon FLIM (2pFLIM) has become one of the most effective techniques for monitoring FRET signals in deep-tissue compartments of living organisms [71]. Levitt et al. performed time-lapse FLIM imaging of intracellular cAMP levels at up to 0.5 frames per second using an Epac-based biosensor, achieving nanometer-level spatial and picosecond-level temporal resolution in live HeLa cells [72]. FLIM-FRET can also be combined with pulsed UV lasers. Kaufmann et al. demonstrated that such integration enhances spatial and temporal resolution, enabling quantitative measurement of DNA damage-related protein interactions in live cells at the single-cell level [65]. Strotmann et al. proposed integrating FLIM-FRET with super-resolution microscopy techniques—such as STED, PALM, and STORM—to overcome diffraction limits and further improve the spatial resolution of intracellular PPI imaging [67]. To address single-point confocal FLIM systems too slow for small-molecule screening, Hirmiz et al. developed a 32 × 32 multiplexed confocal microscope equipped with a time-gated single-photon avalanche diode (SPAD) array, enabling high-speed FLIM acquisition suitable for high-throughput screening applications [73].

FLIM-FRET is hindered by several major limitations. FLIM-FRET requires specialized instrumentation and complex data analysis involving multi-exponential fitting. Photobleaching is another critical issue, particularly severe under pulsed laser illumination, and this problem is further exacerbated when using visible-wavelength lasers. Absolute quantification of FLIM-FRET signals is also impaired by microenvironmental factors.

Although FLIM-FRET requires complex data analysis, the integration of advanced computational methods has transformed FLIM data interpretation, enabling the extraction of meaningful insights from complex lifetime datasets with improved accuracy and efficiency. For example, Betegón-Putze et al. combined FLIM-FRET data with mathematical modeling to explore the regulatory mechanisms of transcription factor complexes involved in plant development [74]. With continued innovations in hardware and computational analysis, FLIM-FRET is poised to play a central role in advancing our understanding of dynamic PPIs across diverse biological contexts.

### 3.4. Single-Molecule FRET

**Figure 7:**
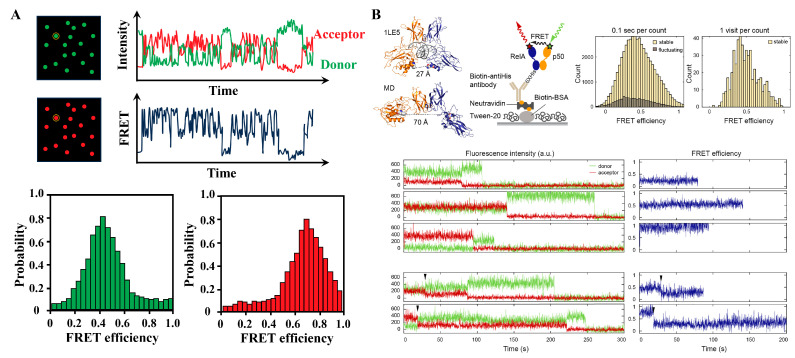
smFRET concept and applications. (**A**) The smFRET reveals the heterogeneity of proteins at the single-molecule level. Example of a two-color smFRET data. Data is acquired in the form of intensities of donor and acceptor, from which apparent FRET efficiency is calculated. (**B**) smFRET revealed a continuum of long-lived conformations for free NF-κB: In the NF-κB/DNA structure, the distance between the labeled positions (spheres) would lead to a high FRET efficiency ∼1; schematic of NF-κB immobilization on a DT20 passivated surface; FRET histogram showing a broad distribution of conformations; FRET histogram constructed by counting each visit to a long-lived state suggested a continuum that cannot be separated into a few groups; representative long-lived states with low, medium and high FRET efficiencies; sudden drops of the donor or acceptor signals to zero indicate single photobleaching events; transitions between long-lived states were captured in a subset of traces. Reproduced from [30] with permission. Copyright 2021, Oxford University Press.

smFRET, as a state-of-the-art technique, is widely used to investigate molecular interactions, conformational changes, and dynamic processes at the single-molecule level. It allows researchers to gain unprecedented insights into the transient and heterogeneous nature of protein interactions, offering a significant advantage in the understanding of biological processes and disease mechanisms. smFRET enables the detection of PPIs at the single-molecule level by isolating individual fluorescent molecules in both space and time, often through surface immobilization in vitro. It relies on advanced optical setups—such as total internal reflection fluorescence (TIRF) microscopy or confocal systems equipped with sensitive detectors (e.g., electron-multiplied charge-coupled device/avalanche photodiode). As the sample is scanned, fluorescence intensities of both donor and acceptor channels are recorded over time, generating time-trace curves that reflect real-time fluctuations in molecular interactions. From these traces, the FRET efficiency is calculated based on the relative fluorescence intensities of the donor and acceptor signals. By compiling data from many individual molecules, researchers can construct a FRET efficiency histogram, which reveals the distribution of interaction states or conformational subpopulations within the system (Figure 7A) [29,75,76]. Chen et al. used smFRET to observe a continuum of NF-κB conformations in both free and DNA-bound states, transitioning on timescales from subseconds to minutes-comparable to physiological binding kinetics and demonstrating how structural dynamics directly influence binding behavior (Figure 7B) [30]. Similar single-molecule approaches have been applied to chaperone systems, such as the Aha1–Hsp90 interaction, revealing how binding stoichiometry regulates function. Mondol et al. combined smFRET, biochemical, and fluorescence anisotropy to systematically investigate the interactions between yeast Aha1 and Hsp90, demonstrating how different stoichiometries of Aha1 binding influence the thermodynamics, kinetics, and function of Hsp90 [77]. Beyond binding kinetics, smFRET has also been instrumental in dissecting stereochemical constraints in disordered complexes. Newcombe et al. investigated the chiral constraints in disordered PPIs. They selected five interacting protein pairs as representative examples, spanning the disorder continuum. By analyzing the natural ligands and their stereochemical mirror images in both free and bound states, they observed that chirality was irrelevant in a fully disordered complex. However, when the interaction depended on the ligand undergoing extensive coupled folding and binding, correct stereochemistry became essential. These findings provide critical insights into the molecular mechanisms underlying complex formation and the application of d-peptides in drug discovery [78]. Such investigations extend to highly charged disordered proteins, where smFRET helps uncover fundamental thermodynamic principles. Chowdhury et al. integrated smFRET, calorimetry, and analytical polymer theory to examine the thermodynamics of high-affinity disordered complexes formed by two highly charged disordered proteins, revealing that the release of counterions constitutes a fundamental entropic contribution to binding [79,80]. Sottini et al. used confocal smFRET spectroscopy to research the interaction between ProTα and H1. Their interaction exhibits a characteristic, the association/dissociation kinetics transition from slow, two-state-like exchange (at low protein concentrations) to fast exchange (at higher concentrations under physiological conditions) [81]. Zosel et al. described a simple and broad strategy for producing proteins for site-specific labeling, intended for smFRET experiments. Additionally, common issues in protein labeling are discussed, along with solutions to avoid them [82]. smFRET has also been pivotal in understanding the dynamics of protein folding, misfolding, and aggregation. For instance, Metskas et al. used smFRET to study the physicochemical properties of intrinsically disordered proteins (IDPs) [83]. This dynamic insight into protein interactions has proven invaluable in areas like enzyme catalysis, signal transduction, and the assembly of protein complexes.

Recent advances in fluorescence microscopy and photon-counting detectors have further enhanced the sensitivity of smFRET, enabling the observation of extremely low-abundance PPIs, including those involving membrane-bound or transiently expressed proteins. For example, Paul et al. utilized a two-photon microscope with the OptiMiS True-Line spectral imaging system to study heterointeractions among nine receptor tyrosine kinase (RTK) pairs, finding that heterodimerization and homodimerization strengths can be similar [84]. Meanwhile, Yu et al. employed inverted ApoTome and wide-field fluorescence microscopy to demonstrate that Bcl-xL inhibits mitophagy by physically interacting with both PINK1 and Parkin, revealing molecular details of this regulatory mechanism [85]. In recent years, multi-color and multi-channel smFRET has greatly enhanced the versatility of the technique. Researchers can now track multiple interactions simultaneously, providing a more comprehensive understanding of complex signaling networks and protein complexes by labeling different proteins with distinct fluorophores. For instance, Cheppali et al. elucidated the chain of events that occur during the disassembly of soluble N-ethylmaleimide sensitive factor attachment protein receptor (NARE) complex by N-ethylmaleimide sensitive factor (NSF) based on single-molecule two- and three-color FRET [86]. This advancement has been especially valuable in studying protein complexes that involve multiple interacting partners.

While smFRET is powerful, it requires precise labeling of proteins with fluorescent probes—inefficient or non-specific labeling, protein size/structure constraints, or endogenous fluorescence can impair data quality. Looking forward, the integration of smFRET with other techniques is expected to provide even greater spatial and temporal resolution for studying protein interactions at the molecular level. Arter et al. mentioned that microfluidic technology can be combined with smFRET for the rapid dilution and analysis of weakly interacting protein complexes [87]. In addition, the development of more sophisticated computational methods for data analysis will likely streamline the interpretation of smFRET data, making it more accessible to a wider range of researchers.

### 3.5. Fluorescence Cross-Correlation Spectroscopy

Fluorescence correlation spectroscopy (FCS) is a highly sensitive technique used to study the dynamic behavior of fluorescently labeled molecules in solution. The measurement is typically performed using confocal microscopy, which enables the precise focusing of a laser beam into a small observation volume (typically on the femtoliter scale). Within this volume, fluorescence intensity fluctuations are recorded as molecules diffuse in and out. These fluctuations are analyzed through an autocorrelation function, which decays over time due to molecular diffusion. By fitting the autocorrelation curve to theoretical diffusion models, key molecular parameters—such as concentration, diffusion coefficients, molecular brightness, and interaction rates—can be determined. FCS is particularly well-suited for low-concentration samples, requiring minimal sample preparation, and it provides real-time, quantitative, and label-free measurements of molecular mobility and dynamics.

**Figure 8:**
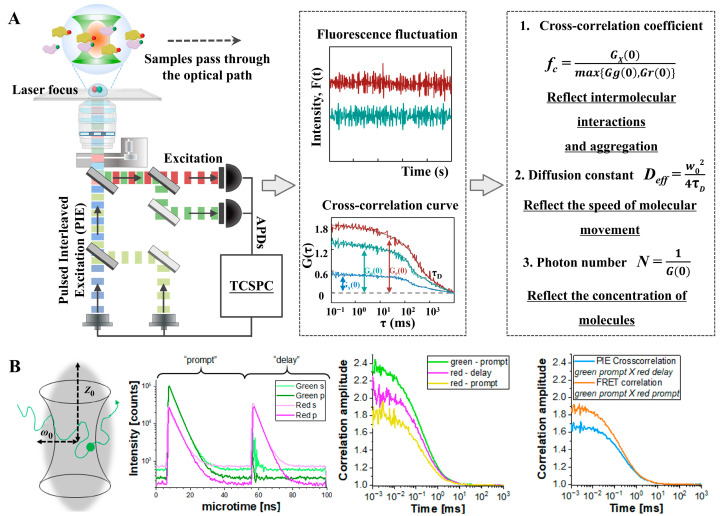
FCCS concept and applications: (**A**) Schematic diagram of the optical path and analysis principle of the PIE-FCCS technology system. Adapted from [31] with permission. Copyright 2023, Science. (**B**) FCCS combined with FRET to study membrane receptor dynamics in live cells. Adapted from [89] with permission. Copyright 2021, Journal of Visualized Experiments.

FCCS is an extension of FCS, in which fluorescence intensity fluctuations from two spectrally distinct channels (e.g., donor and acceptor) are simultaneously recorded and statistically cross-correlated. This allows for the detection of co-diffusing donor–acceptor pairs, providing a robust framework for solution-based smFRET analysis. Therefore, FCCS-FRET is particularly powerful for probing transient and reversible PPIs in live cells, without requiring protein immobilization. By analyzing the cross-correlation, researchers can gather information about the interaction dynamics and the stoichiometry of protein complexes. FCCS-FRET allows the monitoring of dynamic protein complexes in real time, providing insights into the kinetics of protein binding. Many biological processes rely on transient interactions between proteins. Christie et al. introduced pulsed interleaved excitation FCCS (PIE-FCCS), a refined approach that enhances quantification of membrane associations in live cells by minimizing spectral crosstalk. Their study comprehensively reviewed the use of FCCS and PIE-FCCS in analyzing membrane protein interactions [88]. Using PIE-FCCS, Shi et al. investigated the dynamic characteristics of EphA2 receptors in the membranes of live cells. This technique enabled measurement of receptor diffusion behavior, mobility, oligomerization, and spatial density, while also capturing conformational changes within receptor assemblies via fluorescence lifetime data (Figure 8A) [31]. Hemmen et al. developed an experimental approach and data analysis workflow to investigate the dynamics of membrane receptors in live cells. Their method utilized modern fluorescence labeling techniques to monitor receptor behavior in real-time (Figure 8B) [89].

Despite its strengths, FCCS faces several challenges that may limit its broader application. A major limitation is the complexity of data interpretation, particularly in environments with high background fluorescence or rapid molecular diffusion, which can obscure correlation signals. Moreover, the fluorescent properties of labels may be influenced by the target protein structure or by local environmental factors, affecting measurement accuracy. FCCS also requires specialized instrumentation and technical expertise, which may not be readily accessible in all research settings. Another significant challenge is distinguishing specific interactions from nonspecific associations, especially in crowded intracellular environments. Although FCCS offers detailed kinetic and stoichiometric insights, distinguishing true biological interactions from random colocalization remains a technical hurdle.

### 3.6. Summary

In this review, we introduced five widely used FRET-based techniques and their applications in the study of PPIs. Each method offers distinct advantages and limitations, tailored to specific experimental contexts and research needs.

Conventional FRET relies on steady-state fluorescence intensity measurements to detect energy transfer between donor and acceptor fluorophores. It is conceptually simple and allows both qualitative and quantitative assessment of molecular interactions, making it suitable for static binding validation, colocalization analysis, and large-scale screening. However, it is highly susceptible to background fluorescence, spectral crosstalk, and variations in fluorophore concentration, excitation intensity, and dipole orientation.

TR-FRET overcomes these limitations by measuring the donor’s fluorescence lifetime using time-gated detection. It offers a high signal-to-noise ratio, reduced background interference, and enhanced sensitivity, particularly for detecting low-abundance targets. TR-FRET is well-suited for homogeneous assays, high-throughput screening, and proportional binding measurements. Its drawbacks include the need for specialized fluorophores and time-resolved instrumentation.

FLIM-FRET measures changes in donor fluorescence lifetime to determine FRET efficiency, independent of intensity or concentration. This approach enables spatially resolved and highly quantitative analysis of PPIs under physiological conditions. It is particularly useful for live-cell imaging of context-sensitive interactions and mapping subcellular interaction dynamics. However, it requires complex and expensive instrumentation.

smFRET enables the detection of molecular interactions and conformational dynamics at the individual molecule level with nanometer spatial precision and millisecond temporal resolution. It is ideal for probing molecular heterogeneity, transition states, and absolute distances within dynamic complexes. However, smFRET typically requires immobilization, operates in vitro, and demands highly sensitive optical systems, precise labeling strategies, and complex data processing.

FCCS analyzes the co-diffusion of two spectrally distinct fluorophores to quantify molecular interactions in solution or live cells, without requiring immobilization. When combined with FRET (FCCS-FRET), it enables simultaneous measurement of binding dynamics and spatial proximity, offering a powerful platform for quantifying PPIs in live-cell environments. FCCS provides high sensitivity and quantitative capacity without a strict distance constraint, but is limited by instrumentation complexity and challenges in data interpretation, especially in crowded cellular environments.

In comparison, smFRET provides unmatched resolution for studying molecular dynamics but is technically demanding. FCCS-FRET excels in real-time, live-cell quantification of co-diffusion events, though it lacks spatial imaging capabilities. FLIM-FRET delivers high-resolution spatial mapping of PPIs, particularly suited for complex and heterogeneous environments. TR-FRET is optimal for high-throughput, low-background applications, while conventional FRET remains a convenient choice for routine interaction screening. Each technique occupies a unique niche, and their combined application or integration can potentially provide a more comprehensive understanding of PPIs. The choice of method depends on experimental goals, sample type, and desired resolution.

The time scale of PPIs is also a critical factor in selecting appropriate FRET techniques. Transient weak interactions (millisecond scale) require FCCS-FRET with μs-ms temporal resolution [88]. Dynamic regulated interactions (seconds to minutes) are best studied using smFRET or FLIM-FRET, the latter capturing sub-second conformational changes [90]. Steady-state strong interactions (minutes to hours) align with conventional FRET or TR-FRET for minute-level integration of high-affinity complexes [91]. Experimental design must match the technique’s temporal resolution with PPI kinetics.

To support researchers in designing FRET experiments, several valuable online resources have been developed:

- FPbase (https://www.fpbase.org/, accessed on 1 August 2025) is an open access, searchable database for fluorescent proteins. It provides information on protein evolution, sequence mutations, excitation/emission spectra, quantum yield, brightness, photostability, and structural features [92].
- Nikon’s MicroscopyU (https://www.microscopyu.com/, accessed on 19 September 2025) offers comprehensive educational content on the theoretical basis of FRET, selection of fluorescent proteins, and detailed methodological guides for FP-FRET experiments [93].
- Thermo Fisher’s Fluorescence SpectraViewer (https://www.thermofisher.com/order/fluorescence-spectraviewer#!/, accessed on 1 August 2025) is an interactive tool that allows users to compare the spectral properties of fluorophores, adjust excitation sources and filters, and evaluate the compatibility of fluorophores within specific detection setups [94].

Table 2 summarizes the principles, strengths, limitations, and instrumentation of each FRET-based approach discussed in this review.

**Table 2:** FRET-based techniques for PPIs.

Techniques	Principles	Advantages	Limitations	Key Instruments	Critical Fluorophore Parameters and Typical Pair	References
Conventional FRET	Based on the FRET phenomenon, when the donor emission spectrum overlaps with the acceptor absorption spectrum, energy is transferred from the donor to the acceptor, which weakens the donor fluorescence and possibly enhances the acceptor; the acceptor does not necessarily need to be fluorescent.	It is widely used in living cells and allows qualitative and quantitative detection of intermolecular interactions.	It is susceptible to the influence of dye concentration and excitation light intensity.	Fluorescence spectroscopy/microscopy/plate reader	Spectral compatibility, photostability, and biocompatibility. Cy3-Cy5, GFP-RFP.	[37,38,40,41,42,44,45,46,47,48,49,50,84,85,95,96,97]
TR-FRET	The time-resolved technique was used to eliminate the interference of background fluorescence and improve the detection sensitivity of FRET.	With low background and high sensitivity, it is suitable for the detection of low-abundance molecules and can avoid the interference of autofluorescence.	Special markers and time-resolved equipment are required.	Fluorescence lifetime instrument	Long fluorescence lifetime. Tb-Cy5.	[27,51,54,59,60,61,62,63,98]
FLIM-FRET	FRET efficiency is reflected by measuring the donor fluorescence lifetime changes, which are independent of fluorescence intensity.	Offering high quantitative accuracy and environmental robustness.	The equipment is complex; the cost is high.	Fluorescence lifetime imaging microscope	Biocompatibility. CFP-YFP, EGFP-mCherry.	[28,64,65,66,67,68,69,71,72,74,99]
smFRET	Detected at the single-molecule level to observe the energy transfer of individual donor and acceptor molecules and to study the conformational changes and dynamic processes of individual biomolecules.	It can measure the dynamic changes in a single molecule with high precision and avoid the interference of group measurement averaging.	High-sensitivity equipment is required, the technical requirements are high, and data processing is complex.	Total internal reflection fluorescent microscope/ Confocal microscope	Photostability and brightness. Cy3-Cy5, Alexa 488-Alexa 555.	[30,82,83,86]
FCCS-FRET	Normally, FCCS-FRET analyzes the motion correlation of two fluorescently labelled molecules by detecting their diffusion behavior in solution or in cells.	High sensitivity, no need for sample fixation, high quantification capacity, and no distance limitation.	Complex equipment, fluorescent labelling, and solution/homogenised environments only	Confocal microscope integrated with TCSPC	Photostability. EGFP-mCherry, Atto550-Atto647N.	[31,88,89]

## 4. Conclusions

In this review, we introduced the fundamental principles of FRET, including commonly used equations, key considerations for experimental design, as well as major techniques of FRET. We discussed the underlying principles of each FRET modality and conducted a comparative analysis of their applications in PPI research. Through this review, we aim to assist researchers in selecting the most appropriate FRET-based method for their specific experimental objectives.

Significant advancements have been made in both the development of FRET donor–acceptor pairs and in the analytical tools used to interpret FRET data. Novel fluorophore pairs with improved brightness and longer fluorescence lifetimes have greatly enhanced the detection of weak or transient interactions, enabling the study of PPIs involving low-abundance proteins or those expressed in rare cell populations. For instance, Petutschnig et al. established a novel FRET pair composed of mCitrine and mScarlet-I, which offers advantages over earlier systems, particularly in its suitability for analyzing low-abundance proteins in stably transformed plants and for colocalization studies [99]. In parallel, advancements in computational tools have improved the accuracy and depth of FRET data analysis. Software platforms—often in combination with molecular dynamics simulations—allow researchers to model interaction interfaces and predict conformational changes during protein binding. For example, Botti et al. used FRET measurements to determine the distance between the lone tryptophan of human serum albumin (HSA) and a fluorescently labeled miR4749. These distances were subsequently applied in molecular docking and binding free energy calculations to construct a reliable topological model of the HSA–miRNA complex [97].

Looking forward, the integration of super-resolution microscopy and multiplex FRET techniques will allow simultaneous monitoring of multiple PPIs with higher spatial precision and improved detection sensitivity. These innovations will be especially valuable for dissecting complex interaction networks and spatial organization in living cells and tissues. In addition, the incorporation of artificial intelligence and machine learning algorithms into FRET data analysis workflows is expected to streamline large-scale experiments, automate pattern recognition, and enhance interpretation accuracy—thereby accelerating discoveries in systems biology and molecular medicine. Live-cell and in vivo applications of FRET will further deepen our understanding of dynamic PPIs within physiological contexts, bringing FRET technology closer to clinical research and translational medicine. As these technological innovations mature, they will not only expand the capabilities of basic life science research but also open up new avenues in diagnostics, drug development, and personalized medicine.

## Figures and Tables

**Table 1:** PPI techniques: advantages and limitations comparison.

Technique	Under Physiological Conditions	Dynamic Monitoring	Spatial Resolution	Throughput	Quantitative Precision	Label-Free	In Vivo Compatibility	Operational Simplicity	Cost-Effectiveness	Clinical Translation
ELISA	L	△	△	E	△	✗	✗	E	E	E
ITC	L	L	L	L	E	E	✗	L	L	L
GST Pull-down	L	L	L	L	L	L	✗	G	G	L
FP	L	△	△	E	G	✗	✗	E	G	△
SPR	L	G	L	△	E	G	✗	L	L	G
BLI	L	G	L	△	E	G	✗	△	△	G
XL-MS	G	△	G	△	△	L	✗	L	△	△
Y2H	G	△	L	G	L	L	✗	△	E	✗
Co-IP	G	L	L	L	L	L	△	G	E	L
AP-MS	G	L	L	G	△	L	△	△	△	△
LCA	G	△	G	G	△	✗	E	G	G	△
PL	E	△	E	△	△	✗	G	L	L	△
FC	△	L	△	L	L	✗	G	L	L	L
FRET	E	E	E	L	△	✗	E	L	L	△
BiFC	E	△	E	L	L	✗	E	G	△	L
PLA	E	△	E	L	L	✗	E	△	△	E

Excellent: E; Good: G; Limited: L; △: Conditional; ✗: Not applicable. ELISA: enzyme-linked immunosorbent assay, ITC: isothermal titration calorimetry, GST Pull-down: glutathione-S-transferase pull-down, FP: fluorescence polarization, SPR: surface plasmon resonance, BLI: bio-layer interferometry, XL-MS: cross-linking mass spectrometry, Y2H: yeast two-hybrid, Co-IP: co-immunoprecipitation, AP-MS: affinity purification coupled-mass spectrometry, LCA: luciferase complementation assay, PL: proximity labeling, FC: fluorescence co-localization, FRET: Förster resonance energy transfer, BiFC: bimolecular fluorescence complementation, PLA: proximity ligation assay.

## Data Availability

No new data were created or analyzed in this study. Data sharing is not applicable to this article.

## Abbreviations

The following abbreviations are used in this manuscript:

AP-MS	Affinity purification–mass spectrometry
BiFC	Bimolecular fluorescence complementation
BLI	Bio-layer interferometry
BRET	Bioluminescence resonance energy transfer
Co-IP	Co-immunoprecipitation
ELISA	Enzyme-linked immunosorbent assay
FC	Fluorescence co-localization
FCS	Fluorescence correlation spectroscopy
FCCS	Fluorescence cross-correlation spectroscopy
FLIM	Fluorescence lifetime imaging microscopy
FP	Fluorescence polarization
FRET	Förster resonance energy transfer
GST	Glutathione-S-transferase
IDPs	Intrinsically disordered proteins
ITC	Isothermal titration calorimetry
LCA	Luciferase complementation assay
NF-κB	Nuclear factor kappa B
PALM	Photoactivated localization microscopy
PIE	Pulsed interleaved excitation
PL	Proximity labeling
PLA	Proximity ligation assay
PPIs	Protein-protein interactions
RNP	Ribonucleoprotein
smFRET	Single-molecule Förster resonance energy transfer
SPAD	Single-photon avalanche diode
SPR	Surface plasmon resonance
STED	Stimulated emission depletion
STORM	Stochastic optical reconstruction microscopy
TCSPC	Time-correlated single photon counting
TR-FRET	Time-resolved Förster resonance energy transfer
XL-MS	Cross-linking mass spectrometry
Y2H	Yeast two-hybrid

## References

[1] Alberts B. (1998). The cell as a collection of protein machines: Preparing the next generation of molecular biologists. Cell.

[2] Ren H., Ou Q., Pu Q., Lou Y., Yang X., Han Y., Liu S. (2024). Comprehensive review on bimolecular fluorescence complementation and its application in deciphering protein–protein interactions in cell signaling pathways. Biomolecules.

[3] Duan Z., Li K., Duan W., Zhang J., Xing J. (2022). Probing membrane protein interactions and signaling molecule homeostasis in plants by Förster resonance energy transfer analysis. J. Exp. Bot..

[4] Kim D.N., McNaughton A.D., Kumar N. (2024). Leveraging artificial intelligence to expedite antibody design and enhance antibody–antigen interactions. Bioengineering.

[5] Lucero B., Francisco K.R., Liu L.J., Caffrey C.R., Ballatore C. (2023). Protein–protein interactions: Developing small-molecule inhibitors/stabilizers through covalent strategies. Trends Pharmacol. Sci..

[6] Wu D., Li Y., Zheng L., Xiao H., Ouyang L., Wang G., Sun Q. (2023). Small molecules targeting protein–protein interactions for cancer therapy. Acta Pharm. Sin. B.

[7] Tomkins J.E., Manzoni C. (2021). Advances in protein-protein interaction network analysis for Parkinson’s disease. Neurobiol. Dis..

[8] Bruno P.S., Arshad A., Gogu M.-R., Waterman N., Flack R., Dunn K., Darie C.C., Neagu A.-N. (2025). Post-translational modifications of proteins orchestrate all hallmarks of cancer. Life.

[9] Oláh J., Szénási T., Lehotzky A., Norris V., Ovádi J. (2022). Challenges in discovering drugs that target the protein–protein interactions of disordered proteins. Int. J. Mol. Sci..

[10] Tsapras P., Nezis I.P. (2022). A yeast two-hybrid screening identifies novel Atg8a interactors in drosophila. Autophagy.

[11] Tan L., Yammani R.R. (2022). Co-immunoprecipitation-blotting: Analysis of protein-protein interactions. Methods Mol. Biol..

[12] Gnanasekaran P., Pappu H.R. (2023). Detection of protein-protein interactions using glutathione-s-transferase (GST) pull-down assay technique. Methods Mol. Biol..

[13] Gnanasekaran P., Pappu H.R. (2023). Affinity purification-mass spectroscopy (AP-MS) and co-immunoprecipitation (Co-IP) technique to study protein-protein interactions. Methods Mol. Biol..

[14] Greenblatt J.F., Alberts B.M., Krogan N.J. (2024). Discovery and significance of protein-protein interactions in health and disease. Cell.

[15] Akbarzadeh S., Coşkun Ö., Günçer B. (2024). Studying protein–protein interactions: Latest and most popular approaches. J. Struct. Biol..

[16] Cai Z., Wang D., Li Z., Gu M., You Q., Wang L. (2025). The value of coimmunoprecipitation (Co-IP) assays in drug discovery. Expert Opin. Drug Discov..

[17] Arakawa M., Morita E. (2023). Protein pull-down assay using HiBiT-tag-dependent luciferase activity measurement. Bio Protoc..

[18] Richards A.L., Eckhardt M., Krogan N.J. (2021). Mass spectrometry-based protein–protein interaction networks for the study of human diseases. Mol. Syst. Biol..

[19] Tabar M.S., Parsania C., Chen H., Su X.-D., Bailey C.G., Rasko J.E. (2022). Illuminating the dark protein-protein interactome. Cell Rep. Methods.

[20] Yi S., Kim E., Yang S., Kim G., Bae D.W., Son S.Y., Jeong B.G., Ji J.S., Lee H.H., Hahn J.S. (2025). Direct quantification of protein–protein interactions in living bacterial cells. Adv. Sci..

[21] Förster T. (1948). Zwischenmolekulare energiewanderung und fluoreszenz. Ann. Phys..

[22] Lakowicz J. (2006). Principles of Fluorescence Spectroscopy.

[23] Sarkar M., Reshma Raj R., Maliekal T.T. (2024). Finding the partner: FRET and beyond. Exp. Cell Res..

[24] Gong H., Zhang Y., Gao Y., Tian X., Wu P., Wei X., Guo Y. (2023). In vivo precision imaging of vicinal-dithiol-containing proteins by a FRET molecular probe sensitive to protein environment. Chin. Chem. Lett..

[25] Besson B., Eun H., Kim S., Windisch M.P., Bourhy H., Grailhe R. (2022). Optimization of BRET saturation assays for robust and sensitive cytosolic protein-protein interaction studies. Sci. Rep..

[26] Yang F., Qu W., Du M., Mai Z., Wang B., Ma Y., Wang X., Chen T. (2020). Stoichiometry and regulation network of Bcl-2 family complexes quantified by live-cell FRET assay. Cell. Mol. Life Sci..

[27] Tang C., Niu Q., Cicka D., Du Y., Mo X., Fu H. (2021). A time-resolved fluorescence resonance energy transfer screening assay for discovery of protein-protein interaction modulators. STAR Protoc..

[28] Khramtsov Y.V., Ulasov A.V., Slastnikova T.A., Rosenkranz A.A., Lupanova T.N., Georgiev G.P., Sobolev A.S. (2023). Modular nanotransporters delivering biologically active molecules to the surface of mitochondria. Pharmaceutics.

[29] Lerner E., Barth A., Hendrix J., Ambrose B., Birkedal V., Blanchard S.C., Börner R., Sung Chung H., Cordes T., Craggs T.D. (2021). FRET-based dynamic structural biology: Challenges, perspectives and an appeal for open-science practices. Elife.

[30] Chen W., Lu W., Wolynes P.G., Komives E.A. (2021). Single-molecule conformational dynamics of a transcription factor reveals a continuum of binding modes controlling association and dissociation. Nucleic Acids Res..

[31] Shi X., Lingerak R., Herting C.J., Ge Y., Kim S., Toth P., Wang W., Brown B.P., Meiler J., Sossey-Alaoui K. (2023). Time-resolved live-cell spectroscopy reveals EphA2 multimeric assembly. Science.

[32] Főrster T. (1959). Transfer mechanisms of electronic excitation. Discuss. Faraday Soc..

[33] Valeur B. (2001). Molecular Fluorescence: Principles and Applications.

[34] Algar W.R., Hildebrandt N., Vogel S.S., Medintz I.L. (2019). FRET as a biomolecular research tool—understanding its potential while avoiding pitfalls. Nat. Methods.

[35] Li Y.X., Xie D.T., Yang Y.X., Chen Z., Guo W.Y., Yang W.C. (2022). Development of small-molecule fluorescent probes targeting enzymes. Molecules.

[36] Jiang X., Yang R., Lei X., Xue S., Wang Z., Zhang J., Yan L., Xu Z., Chen Z., Zou P. (2024). Design, synthesis, application and research progress of fluorescent probes. J. Fluoresc..

[37] Soleja N., Mohsin M. (2024). Exploring the landscape of FRET-based molecular sensors: Design strategies and recent advances in emerging applications. Biotechnol. Adv..

[38] Kong L., Jiang D., He C., Xia J., Wei H., Zhou L., Peng H. (2020). Tg ROP18 targets IL20RB for host-defense-related-STAT3 activation during Toxoplasma gondii infection. Parasit. Vectors.

[39] Zhou Y., Wang Y., Li J., Liang J. (2021). In vivo FRET-FLIM reveals ER-specific increases in the ABA level upon environmental stresses. Plant Physiol..

[40] Fang C., Huang Y., Zhao Y. (2023). Review of FRET biosensing and its application in biomolecular detection. Am. J. Transl. Res..

[41] Hoshino N., Kanadome T., Takasugi T., Itoh M., Kaneko R., Inoue Y.U., Inoue T., Hirabayashi T., Watanabe M., Matsuda T. (2023). Visualization of trans homophilic interaction of clustered protocadherin in neurons. Proc. Natl. Acad. Sci. USA.

[42] Verma A.K., Noumani A., Yadav A.K., Solanki P.R. (2023). FRET based biosensor: Principle applications recent advances and challenges. Diagnostics.

[43] Sowa S.T., Vela-Rodríguez C., Galera-Prat A., Cázares-Olivera M., Prunskaite-Hyyryläinen R., Ignatev A., Lehtiö L. (2020). A FRET-based high-throughput screening platform for the discovery of chemical probes targeting the scaffolding functions of human tankyrases. Sci. Rep..

[44] Wan Q., Mouton S.N., Veenhoff L.M., Boersma A.J. (2022). A FRET-based method for monitoring structural transitions in protein self-organization. Cell Rep. Methods.

[45] Han K., Suh J.S., Choi G., Jang Y.K., Ahn S., Lee Y., Kim T.J. (2025). Novel FRET-based biosensors for real-time monitoring of estrogen receptor dimerization and translocation dynamics in living cells. Adv. Sci..

[46] Glöckner N., zur Oven-Krockhaus S., Rohr L., Wackenhut F., Burmeister M., Wanke F., Holzwart E., Meixner A.J., Wolf S., Harter K. (2022). Three-fluorophore FRET enables the analysis of ternary protein association in living plant cells. Plants.

[47] Stoneman M.R., Raicu N., Biener G., Raicu V. (2020). Fluorescence-based methods for the study of protein-protein interactions modulated by ligand binding. Curr. Pharm. Des..

[48] Kroeck K.G., Qiu W., Catalano C., Trinh T.K.H., Guo Y. (2020). Native cell membrane nanoparticles system for membrane protein-protein interaction analysis. J. Vis. Exp..

[49] Adhikari D.P., Stoneman M.R., Raicu V. (2025). Impact of photobleaching of fluorescent proteins on FRET measurements under two-photon excitation. Spectrochim. Acta A.

[50] Shu X. (2020). Imaging dynamic cell signaling in vivo with new classes of fluorescent reporters. Curr. Opin. Chem. Biol..

[51] Kieffer C., Jourdan J.P., Jouanne M., Voisin-Chiret A.S. (2020). Noncellular screening for the discovery of protein–protein interaction modulators. Drug Discov. Today.

[52] Yu L., Feng N., Fu W., Huang X., Li X., Xin X., Hao J., Li H. (2025). Near-infrared organic ultralong room-temperature phosphorescence materials constructed via multiple phosphorescence resonance energy transfer. Adv. Opt. Mater..

[53] Larsen E.K., Abreu-Blanco M., Rabara D., Stephen A.G. (2024). KRAS4b: RAF-1 homogenous time-resolved fluorescence resonance energy transfer assay for drug discovery. KRAS: Methods and Protocols.

[54] Cicka D., Niu Q., Qui M., Qian K., Miller E., Fan D., Mo X., Ivanov A.A., Sarafianos S.G., Du Y. (2023). TMPRSS2 and SARS-CoV-2 SPIKE interaction assay for uHTS. J. Mol. Cell Biol..

[55] Funato Y., Mimura M., Nunomura K., Lin B., Fujii S., Haruta J., Miki H. (2024). Development of a high-throughput screening system targeting the protein-protein interactions between PRL and CNNM. Sci. Rep..

[56] Yang J., Basu S., Hu L. (2022). Design, synthesis, and structure–activity relationships of 1, 2, 3, 4-tetrahydroisoquinoline-3-carboxylic acid derivatives as inhibitors of the programmed cell death-1 (PD-1)/programmed cell death-ligand 1 (PD-L1) immune checkpoint pathway. Med. Chem. Res..

[57] Wang T., Cai S., Wang M., Zhang W., Zhang K., Chen D., Li Z., Jiang S. (2021). Novel biphenyl pyridines as potent small-molecule inhibitors targeting the programmed cell death-1/programmed cell death-ligand 1 interaction. J. Med. Chem..

[58] Lee S., Ali A.R., Abed D.A., Nguyen M.U., Verzi M.P., Hu L. (2024). Structural modification of C2-substituents on 1,4-bis(arylsulfonamido)benzene or naphthalene-N,N′-diacetic acid derivatives as potent inhibitors of the Keap1-Nrf2 protein-protein interaction. Eur. J. Med. Chem..

[59] Lee S., Abed D.A., Beamer L.J., Hu L. (2021). Development of a homogeneous time-resolved fluorescence resonance energy transfer (TR-FRET) assay for the inhibition of Keap1–Nrf2 protein–protein interaction. SLAS Discov..

[60] Abed D.A., Ali A.R., Lee S., Nguyen M.-U., Verzi M.P., Hu L. (2023). Optimization of the C2 substituents on the 1, 4-bis (arylsulfonamido) naphthalene-N, N′-diacetic acid scaffold for better inhibition of Keap1-Nrf2 protein-protein interaction. Eur. J. Med. Chem..

[61] Harada M., Nagai J., Kurata R., Cui X., Isagawa T., Semba H., Yoshida Y., Takeda N., Maemura K., Yonezawa T. (2021). Establishment of novel protein interaction assays between Sin3 and REST using surface plasmon resonance and time-resolved fluorescence energy transfer. Int. J. Mol. Sci..

[62] Chen T., Pham H., Mohamadi A., Miller L.W. (2020). Single-chain lanthanide luminescence biosensors for cell-based imaging and screening of protein-protein interactions. iScience.

[63] Tang C., Mo X., Niu Q., Wahafu A., Yang X., Qui M., Ivanov A.A., Du Y., Fu H. (2021). Hypomorph mutation-directed small-molecule protein-protein interaction inducers to restore mutant SMAD4-suppressed TGF-β signaling. Cell Chem. Biol..

[64] Datta R., Heaster T.M., Sharick J.T., Gillette A.A., Skala M.C. (2020). Fluorescence lifetime imaging microscopy: Fundamentals and advances in instrumentation, analysis, and applications. J. Biomed. Opt..

[65] Kaufmann T., Herbert S., Hackl B., Besold J.M., Schramek C., Gotzmann J., Elsayad K., Slade D. (2020). Direct measurement of protein-protein interactions by FLIM-FRET at UV laser-induced DNA damage sites in living cells. Nucleic Acids Res..

[66] Melle C., Hoffmann B., Wiesenburg A., Biskup C. (2024). FLIM-FRET-based analysis of S100A11/annexin interactions in living cells. FEBS Open Bio.

[67] Strotmann V.I., Stahl Y. (2022). Visualization of in vivo protein-protein interactions in plants. J. Exp. Bot..

[68] Krieger N., Pastryk K.-F., Forchhammer K., Kolukisaoglu Ü. (2021). Arabidopsis PII proteins form characteristic foci in chloroplasts indicating novel properties in protein interaction and degradation. Int. J. Mol. Sci..

[69] Fahim L.E., Marcus J.M., Powell N.D., Ralston Z.A., Walgamotte K., Perego E., Vicidomini G., Rossetta A., Lee J.E. (2025). Fluorescence lifetime sorting reveals tunable enzyme interactions within cytoplasmic condensates. J. Cell Biol..

[70] Eckenstaler R., Benndorf R.A. (2021). A combined acceptor photobleaching and donor fluorescence lifetime imaging microscopy approach to analyze multi-protein interactions in living cells. Front. Mol. Biosci..

[71] Murakoshi H. (2021). Optogenetic Imaging of Protein Activity Using Two-Photon Fluorescence Lifetime Imaging Microscopy.

[72] Levitt J.A., Poland S.P., Krstajic N., Pfisterer K., Erdogan A., Barber P.R., Parsons M., Henderson R.K., Ameer-Beg S.M. (2020). Quantitative real-time imaging of intracellular FRET biosensor dynamics using rapid multi-beam confocal FLIM. Sci. Rep..

[73] Hirmiz N., Tsikouras A., Osterlund E.J., Richards M., Andrews D.W., Fang Q. (2020). Highly multiplexed confocal fluorescence lifetime microscope designed for screening applications. IEEE J. Sel. Top. Quantum Electron..

[74] Betegón-Putze I., Mercadal J., Bosch N., Planas-Riverola A., Marquès-Bueno M., Vilarrasa-Blasi J., Frigola D., Burkart R.C., Martínez C., Conesa A. (2021). Precise transcriptional control of cellular quiescence by BRAVO/WOX5 complex in Arabidopsis roots. Mol. Syst. Biol..

[75] Nettels D., Galvanetto N., Ivanović M.T., Nüesch M., Yang T., Schuler B. (2024). Single-molecule FRET for probing nanoscale biomolecular dynamics. Nat. Rev. Phys..

[76] Agam G., Gebhardt C., Popara M., Mächtel R., Folz J., Ambrose B., Chamachi N., Chung S.Y., Craggs T.D., de Boer M. (2023). Reliability and accuracy of single-molecule FRET studies for characterization of structural dynamics and distances in proteins. Nat. Methods.

[77] Mondol T., Silbermann L.M., Schimpf J., Vollmar L., Hermann B., Tych K.K., Hugel T. (2023). Aha1 regulates Hsp90’s conformation and function in a stoichiometry-dependent way. Biophys. J..

[78] Newcombe E.A., Due A.D., Sottini A., Elkjær S., Theisen F.F., Fernandes C.B., Staby L., Delaforge E., Bartling C.R.O., Brakti I. (2024). Stereochemistry in the disorder-order continuum of protein interactions. Nature.

[79] Chowdhury A., Borgia A., Ghosh S., Sottini A., Mitra S., Eapen R.S., Borgia M.B., Yang T., Galvanetto N., Ivanović M.T. (2023). Driving forces of the complex formation between highly charged disordered proteins. Proc. Natl. Acad. Sci. USA.

[80] Stelzl L.S., Pietrek L.M., Holla A., Oroz J., Sikora M., Köfinger J., Schuler B., Zweckstetter M., Hummer G. (2022). Global structure of the intrinsically disordered protein tau emerges from its local structure. JACS Au.

[81] Sottini A., Borgia A., Borgia M.B., Bugge K., Nettels D., Chowdhury A., Heidarsson P.O., Zosel F., Best R.B., Kragelund B.B. (2020). Polyelectrolyte interactions enable rapid association and dissociation in high-affinity disordered protein complexes. Nat. Commun..

[82] Zosel F., Holla A., Schuler B. (2022). Labeling of proteins for single-molecule fluorescence spectroscopy. Methods Mol. Biol..

[83] Metskas L.A., Rhoades E. (2020). Single-molecule FRET of intrinsically disordered proteins. Annu. Rev. Phys. Chem..

[84] Paul M.D., Grubb H.N., Hristova K. (2020). Quantifying the strength of heterointeractions among receptor tyrosine kinases from different subfamilies: Implications for cell signaling. J. Biol. Chem..

[85] Yu S., Du M., Yin A., Mai Z., Wang Y., Zhao M., Wang X., Chen T. (2020). Bcl-xL inhibits PINK1/Parkin-dependent mitophagy by preventing mitochondrial Parkin accumulation. Int. J. Biochem. Cell Biol..

[86] Cheppali S.K., Li C., Xing W., Sun R., Yang M., Xue Y., Lu S.-Y., Yao J., Sun S., Chen C. (2025). Single-molecule two-and three-colour FRET studies reveal a transition state in SNARE disassembly by NSF. Nat. Commun..

[87] Arter W.E., Levin A., Krainer G., Knowles T.P. (2020). Microfluidic approaches for the analysis of protein–protein interactions in solution. Biophys. Rev..

[88] Christie S., Shi X., Smith A.W. (2020). Resolving membrane protein–protein interactions in live cells with pulsed interleaved excitation fluorescence cross-correlation spectroscopy. Acc. Chem. Res..

[89] Hemmen K., Choudhury S., Friedrich M., Balkenhol J., Knote F., Lohse M.J., Heinze K.G. (2021). Dual-color fluorescence cross-correlation spectroscopy to study protein-protein interaction and protein dynamics in live cells. J. Vis. Exp..

[90] Harkes R., Kukk O., Mukherjee S., Klarenbeek J., van den Broek B., Jalink K. (2021). Dynamic FRET-FLIM based screening of signal transduction pathways. Sci. Rep..

[91] Rajapakse H.E., Gahlaut N., Mohandessi S., Yu D., Turner J.R., Miller L.W. (2010). Time-resolved luminescence resonance energy transfer imaging of protein–protein interactions in living cells. Proc. Natl. Acad. Sci. USA.

[92] Lambert T.J. (2019). FPbase: A community-editable fluorescent protein database. Nat. Methods.

[93] Fellers T., Davidson M. MicroscopyU, The Source for Microscopy Education-Linear Measurements (Micrometry). NIKON Instruments Inc.. https://www.microscopyu.com.

[94] Scientific T. (2016). Fluorescence Spectraviewer.

[95] Shkhair A.I., Madanan A.S., Varghese S., Abraham M.K., Indongo G., Rajeevan G., Kala A.B., Abbas S.M., George S. (2024). Bovine serum albumin-capped fluorescent copper nanocluster incorporated with 2D-molybdenum disulfide nanosheets as a FRET-based immune probe for the “Turn-On” detection of cTnT. ACS Appl. Bio Mater..

[96] Pope J.R., Johnson R.L., Jamieson W.D., Worthy H.L., Kailasam S., Ahmed R.D., Taban I., Auhim H.S., Watkins D.W., Rizkallah P.J. (2021). Association of fluorescent protein pairs and its significant impact on fluorescence and energy transfer. Adv. Sci..

[97] Botti V., Marrone S., Cannistraro S., Bizzarri A.R. (2022). Interaction between miR4749 and human serum albumin as revealed by fluorescence, FRET, atomic force spectroscopy and computational modelling. Int. J. Mol. Sci..

[98] Brink H.J., Riemens R., Thee S., Beishuizen B., da Costa Pereira D., Wijtmans M., de Esch I., Smit M.J., de Boer A.H. (2022). Fragment screening yields a small-molecule stabilizer of 14-3-3 dimers that modulates client protein interactions. ChemBioChem.

[99] Petutschnig E.K., Pierdzig L., Mittendorf J., Niebisch J.M., Lipka V. (2024). A novel fluorescent protein pair facilitates FLIM-FRET analysis of plant immune receptor interaction under native conditions. J. Exp. Bot..

